# Analysis of antibiotic treatment of children in a Shanghai tertiary hospital based on point prevalence surveys

**DOI:** 10.1186/s12879-020-05542-1

**Published:** 2020-10-29

**Authors:** Jiang-Jiang Xu, Jie Gao, Jun-Hua Guo, Li-Li Song

**Affiliations:** Departments of Infection Control, Shanghai Children’s Hospital, Shanghai Jiaotong University, Shanghai, China

**Keywords:** Antibiotic, Third-generation cephalosporin, Carbapenem, Prophylaxis, Children

## Abstract

**Background:**

Misuse and overuse of antibiotics by physicians in the treatment of children is common in China. This study aimed to reveal the overall use of antibiotics to treat children hospitalized in four types of pediatric wards.

**Methods:**

Seven independent point prevalence surveys (PPSs) were conducted in Shanghai Children’s Hospital of Shanghai Jiao Tong University over the period 2012 to 2018. Pediatric ward types were defined general pediatric medical, pediatric surgical, pediatric intensive care units (PICU), and neonatal.

**Results:**

A total of 3975 pediatric patients were included in the study, of which 63.9% received at least one dose antibiotic. The top five classes of antibiotics administered were cephalosporins (43.8%, *n* = 1743), penicillins (13.2%, *n* = 526), carbapenems (8.7%, *n* = 347), nitroimidazoles (7.1%, *n* = 281) and macrolides (6.5%, *n* = 257). The five most commonly used generic antibiotics were cefuroxime (14.9%, *n* = 594), ceftriaxone (9.7%, *n* = 387), cefotaxime (9.0%, *n* = 358), meropenem (8.1%, *n* = 320) and ampicillin/sulbactam (6.0%, *n* = 239). Meropenem was among top five antibiotics prescribed in the general pediatric, PICU and neonatal wards and sixth in the pediatric surgical wards. Of all children on antibiotics, 23.4% received prophylactic treatment, and prophylaxis accounted for 68.1% of indications for treatment in the pediatric surgical wards.

**Conclusions:**

Given that over-treatment with third-generation cephalosporins and carbapenems has been associated with treatment-resistant infections, the prescription of these drugs should be strictly controlled and monitored, and measures should be taken to improve the management of surgical prophylaxis in hospitalized children in China.

## Background

China has the largest population in the world and also uses a large quantity of antibiotics. A study conducted in 784 community health institutions in 28 cities across China showed that China’s community health institutions prescribed many more antibiotics and more injections than the WHO’s recommendations [1]. Children are usually more susceptible to infections due to their underdeveloped immune system. In China, for lack of knowledge and poor understanding of antibiotics usage among parents [2], administration of antibiotics by parents in the absence of a physician’s advice is a common phenomenon in the community [3, 4]. In hospitals, physicians’ prescriptions for antibiotics are affected not only by their clinical judgment but also by the economic policy and doctor-patient relationships in China. In a survey of doctors in China, Xia and coworkers found that, one fifth to one third of doctors suggest patients self-administer antibiotics in hospitals [5]. Furthermore, in an effort to make treatment quick and effective, doctors might feel pressure to prescribe the most powerful antibiotics for children in hospitals even when not needed [6]. Therefore, hospitals are undoubtedly the most important source of antimicrobial overuse in the treatment of children. A recent study conducted in a Chinese pediatric hospital revealed that 59.6% patients received inappropriate empirical therapy [7]. In a US study, 44% of children undergoing elective clean-contaminated and clean surgical procedures with foreign body implantation received inappropriate prophylaxis [8]. Therefore, the inappropriate use of antimicrobial drugs in hospitals is a problem not restricted to China.

Misuse and overuse of antibiotics causes not only the most common side effects such as antibiotic-associated diarrhea and antibiotic allergic reactions but also higher healthcare costs [9]. If the patient’s inflammation is caused by viral infection, anti-inflammatory effects are often not achieved with antibiotics, and even more serious consequences can result. Van Houten et al. found that children admitted to the PICU were more often to receive antibiotics for viral respiratory tract infections [10]. A recent study indicate a significant association between irrational use of antibiotics and death in children [11]. More troubling, antibiotic resistance has become an important public health issue worldwide. The correlation between overuse of broad-spectrum antibiotics and bacterial resistance has been confirmed in multiple studies [12–15].

This study analyzes the results of seven repeated point prevalence surveys (PPSs) on antibiotic usage carried out in Shanghai Children’s Hospital from 2012 to 2018. We aimed to reveal the general situation of antibiotic prescription practices, including antibiotic number, type of antibiotic agents, antibiotic prescription rates and the indications for antibiotic treatment (therapy, prophylaxis) among hospitalized children in four main types of pediatric wards.

## Methods

### Study settings

The study was conducted in Shanghai Children’s Hospital of Shanghai Jiao Tong University, a tertiary level 800-bed hospital with all medical specialties available, including PICU, neonatal intensive care unit (NICU), neonatology, hematology oncology, respiratory, gastroenterology, nephrology, endocrinology, cardiology, neurology, neurosurgery, cardiothoracic surgery, general surgery, urology, pediatric orthopedics, otolaryngology, and ophthalmology. Data was collected from all wards of the hospital.

### Study design and participants

A one-day PPS on antibiotic treatment of hospitalized children was performed in Shanghai Children’s Hospital. A total of seven PPSs were conducted during the period 2012 to 2018 (once a year in the months of November or December). The survey included all admitted patients present in the ward at 8:00 am on the day of the survey. Patients discharged on the survey day were included, and newly admitted patients were excluded. The total number of patients included in the study was 3975. The number of participants per survey day was as follows: 376 (2012-11-13), 399 (2013-11-20), 578 (2014-11-12), 643 (2015-12-10), 575 (2016-11-30), 684 (2017-12-12) and 720 (2018-12-11). One pediatrician and one nurse in each ward were responsible for investigating and registering the use of antibiotics in the hospitalized children. For all participants, data about gender, age, clinical department, disease diagnoses, and antibiotic treatment characteristics including the categories of antibiotics prescribed, the reasons for prescribing and combinations antibacterial medication were collected. In addition, surgical inpatient records included the surgical site and time of surgery. The study was approved by the Research Ethics Committee of Shanghai Children’s Hospital of Shanghai Jiao Tong University (Approval No: 2020R074-E01). Our team has obtained administrative permissions to access the data used in the study.

### Statistical analysis

Microsoft Excel 2007 (Microsoft Corporation, WA, United States of America) and SPSS 16.0 statistical package (SPSS Inc., Chicago, IL, USA) were used. The categorical variables were presented as counts and percentages. Statistical significance for the differences between groups was assessed using a chi-square test and a two-sided *P* value < 0.05 was considered to be statistically significant.

## Results

### Study population

A total of 3975 patients (1546 girls and 2429 boys) were hospitalized during the seven PPSs. The median age of the cohort was 2 (IQR: 0.23,5.00) years, with a minimum of 1 h and a maximum age of 17 years. Patients were in four main pediatric ward types: general pediatric medical (38.1%, *n* = 1514), pediatric surgical (36.0%, *n* = 1430), PICU (5.0%, *n* = 200) and neonatal wards (20.9%, *n* = 831). Neonatal wards included the NICU, premature, and a general neonatal medical wards. The demographic characteristics and disease diagnoses of the patients included in the current study are summarized in Table 1.

**Table 1:** Baseline characteristics of patients

Characteristics	Groups	Number	Composition ratio (%)
Sex	Male	2428	61.1
	Female	1547	38.9
Age in years	< 1	1449	36.5
	1–5	1654	41.6
	6–10	614	15.4
	≥11	258	6.5
Diagnosis	Surgical problem	1231	31.0
	Neonatal disease	831	20.9
	Pneumonia^a^	577	14.5
	Oncologic	251	6.3
	Congenital heart disease	155	3.9
	Diseases of the immune system	148	3.7
	Acute infection^b^	138	3.5
	Leukemia	141	3.5
	Chronic kidney disease	101	2.5
	Diseases of the digestive system	92	2.3
	Neurological and mental illness	73	1.8
	Other respiratory disease^c^	64	1.6
	Other hematologic disease ^d^	49	1.2
	Sepsis	45	1.1
	Genetic and metabolic disease	43	1.1
	Cardiovascular diseases	19	0.5
	Other/unknown	17	0.4

Note:^a^excluding neonatal pneumonia; ^b^excluding pneumonia and sepsis; ^c^respiratory diseases except pneumonia; ^d^ hematologic diseases except leukemia

### Types and prevalence of antibiotic treatment

A total of 54 different antibiotics were identified in the analysis and were classified into the following 18 categories: cephalosporins, penicillins (including combinations with beta-lactamase inhibitors), carbapenems, nitroimidazoles, macrolides, glycopeptides, lincosamides, cephalomycins, sulfonamides, linezolid, aminoglycosides, fosfomycin, furans, tetracyclines, glycylcyclines, quinolones, aztreonam, and antifungals. Overall, 2541 (63.9%) children received at least one dose of an antibiotic during the seven PPSs. The top five classes of drugs prescribed were cephalosporins (43.8%, *n* = 1743), penicillin-based drugs (13.2%, *n* = 526), carbapenems (8.7%, *n* = 347), nitroimidazoles (7.1%, *n* = 281) and macrolides (6.5%, *n* = 257). Among cephalosporins, third-generation drugs represented 59.4% (*n* = 1035) of prescriptions, while the second-generation drugs accounted for 40.3% (*n* = 702) of prescriptions. The combination of penicillins plus β-lactamase inhibitors accounted for 86.69% (456/526) of penicillin-based prescriptions. Carbapenems were the third most frequently prescribed antibiotic class in our study, with meropenem the most frequently used in this class. The five most commonly used generic antibiotics were cefuroxime (14.9%, *n* = 594), ceftriaxone (9.7%, *n* = 387), cefotaxime (9.0%, *n* = 358), meropenem (8.1%, *n* = 320) and ampicillin/sulbactam (6.0%, *n* = 239) (Table 2).

**Table 2:** Types of antibiotic agents and antibiotic prescribing rates in four types of pediatric wards

Antibiotic classes	Number of Participants [n(%)] (*n* = 3975)	Antibiotic agents	Number of Participants [n (%)] (*n* = 3975)	Prophylaxis ^a^ [n (%)] (*n* = 648)	Therapy ^a^ [n (%)] (*n* = 1947)	General pediatric medical wards (n = 1514)	Pediatric surgical wards (n = 1430)	PICU (n = 200)	Neonatal wards (n = 831)
Cephalosporins	1743 (43.8)	cefuroxime	594 (14.9)	297 (45.8)	301 (15.5)	257 (17.0)	306 (21.4)	30 (15.0)	1 (0.1)
		ceftriaxone	387 (9.7)	81 (12.5)	315 (16.2)	255 (16.8)	111 (7.8)	9 (4.5)	12 (1.4)
		cefotaxime	358 (9.0)	24 (3.7)	344 (17.7)	32 (2.1)	8 (0.6)	8 (4.0)	310 (37.3)
		cefoperazone/sulbactam	236 (5.9)	54 (8.3)	189 (9.7)	73 (4.8)	105 (7.3)	46 (23.0)	12 (1.4)
		cefonicid	49 (1.2)	37 (5.7)	13 (0.7)	11 (0.7)	37 (2.6)	1 (0.5)	0
		cefotiam	33 (0.8)	21 (3.2)	12 (0.6)	11 (0.7)	22 (1.5)	0	0
		ceftazidime	29 (0.7)	7 (1.1)	22 (1.1)	3 (0.2)	14 (1.0)	1 (0.5)	11 (1.3)
		cefaclor	26 (0.7)	18 (2.8)	8 (0.4)	5 (0.3)	19 (1.3)	0	2 (0.2)
		cefixime	22 (0.6)	8 (1.2)	16 (0.8)	6 (0.4)	5 (0.3)	3 (1.5)	8 (1.0)
		cefepime	6 (0.2)	0	6 (0.3)	5 (0.3)	1 (0.1)	0	0
		cefdinir	3 (0.1)	0	3 (0.2)	3 (0.2)	0	0	0
Penicillins	526 (13.2)	ampicillin/sulbactam	239 (6.0)	33 (5.1)	212 (10.9)	27 (1.8)	15 (1.0)	29 (14.5)	168 (20.2)
		amoxycillin/clavulanic acid	113 (2.8)	0	113 (5.8)	104 (6.9)	0	4 (2.0)	5 (0.6)
		ticarcillin/clavulanic acid	99 (2.5)	15 (2.3)	85 (4.4)	1 (0.1)	35 (2.4)	0	63 (7.6)
		penicillin	59 (1.5)	3 (0.5)	56 (2.9)	36 (2.4)	2 (0.1)	0	21 (2.5)
		amoxicillin/sulbactam	5 (0.1)	0	5 (0.3)	3 (0.2)	0	0	2 (0.2)
		mezlocillin	5 (0.1)	0	5 (0.3)	0	0	0	5 (0.6)
		piperacillin	4 (0.1)	1 (0.2)	4 (0.2)	0	3 (0.3)	0	1 (0.1)
		oxacillin	1 (0.0)	0	1 (0.05)	0	1 (0.1)	0	0
		cloxacillin	1 (0.0)	0	1 (0.05)	0	0	0	1 (0.1)
Carbapenems	347 (8.7)	meropenem	320 (8.1)	32 (4.9)	298 (15.3)	89 (5.9)	69 (4.8)	47 (23.5)	115 (13.8)
		imipenem/cilastatin	27 (0.7)	1 (0.2)	27 (1.4)	17 (1.1)	3 (0.2)	6 (3.0)	1 (0.1)
Nitroimidazoles	281 (7.1)	ornidazole	159 (4.0)	90 (13.9)	76 (3.9)	4 (0.3)	154 (10.8)	1 (0.5)	0
		metronidazole	121 (3.0)	58 (9.0)	66 (3.4)	6 (0.4)	89 (6.2)	2 (1.0)	24 (2.9)
		tinidazole	1 (0.0)	1 (0.2)	0	0	1 (0.1)	0	0
Macrolides	257 (6.5)	azithromycin	220 (5.5)	0	220 (11.3)	195 (12.9)	3 (0.2)	22 (11.0)	0
		erythromycin cyclic	22 (0.6)	0	22 (1.1)	9 (0.6)	0	9 (4.5)	4 (0.5)
		acetyl midecamycin	11 (0.3)	0	11 (0.6)	8 (0.5)	1 (0.1)	2 (1.0)	0
		clarithromycin	2 (0.1)	0	2 (0.1)	2 (0.1)	0	0	0
		roxithromycin	1 (0.0)	0	1 (0.05)	0	0	0	1 (0.1)
		acetylspiramycin	1 (0.0)	0	1 (0.05)	0	0	0	1 (0.1)
Glycopeptides	124 (3.1)	vancomycin	115 (2.9)	14 (2.2)	104 (5.3)	45 (3.0)	32 (2.2)	15 (7.5)	23 (2.8)
		demethyl vancomycin	8 (0.2)	0	8 (0.4)	2 (0.1)	4 (0.3)	0	2 (0.2)
		teicoplanin	1 (0.0)	0	1 (0.05)	0	1 (0.1)	0	0
Lincosamides	30 (0.8)	clindamycin	30 (0.8)	15 (2.3)	15 (0.8)	1 (0.1)	29 (2.0)	0	0
Cephalomycins	26 (0.7)	cefoxitin	19 (0.5)	15 (2.3)	4 (0.2)	1 (0.1)	18 (1.3)	0	0
		cefotetan	6 (0.2)	1 (0.2)	0	6 (0.4)	0	0	0
		cefmetazole	1 (0.0)	0	6 (0.3)	1 (0.1)	0	0	0
Sulfonamides	23 (0.6)	compound sulfamethoxazole	23 (0.6)	6 (0.9)	19 (1.0)	22 (1.5)	0	1 (0.5)	0
Linezolid	16 (0.4)	linezolid	16 (0.4)	2 (0.3)	15 (0.8)	11 (0.7)	3 (0.2)	2 (1.0)	0
Aminoglycosides	12 (0.3)	isepamicin	8 (0.2)	1 (0.2)	8 (0.4)	3 (0.2)	0	5 (2.5)	0
		streptomycin	2 (0.1)	0	2 (0.1)	0	2 (0.1)	0	0
		amikacin	2 (0.1)	0	2 (0.1)	0	0	1 (0.5)	1 (0.1)
Fosfomycin	9 (0.2)	fosfomycin	9 (0.2)	1 (0.2)	9 (0.5)	1 (0.1)	1 (0.1)	7 (3.5)	0
Furans	5 (0.1)	nitrofurantoin	5 (0.1)	0	5 (0.3)	0	2 (0.1)	3 (1.5)	0
Tetracyclines	4 (0.1)	minocycline	4 (0.1)	1 (0.2)	4 (0.2)	3 (0.2)	0	1 (0.5)	0
Glycylcyclines	2 (0.1)	tigecycline	2 (0.1)	1 (0.2)	2 (0.1)	1 (0.1)	0	1 (0.5)	0
Quinolones	2 (0.1)	norfloxacin	1 (0.0)	1 (0.2)	0	0	1 (0.1)	0	0
		ciprofloxacin	1 (0.0)	1 (0.2)	1 (0.05)	0	0	1 (0.5)	0
Aztreonam	1 (0.0)	aztreonam	1 (0.0)	0	1 (0.05)	0	0	0	1 (0.1)
Antifungals	92 (2.3)	fluconazole	43 (1.1)	3 (0.5)	41 (2.1)	35 (2.3)	0	3 (1.5)	5 (0.6)
		voriconazole	23 (0.6)	4 (0.6)	23 (1.18)	13 (0.9)	1 (0.1)	6 (3.0)	3 (0.4)
		itraconazole	21 (0.5)	4 (0.6)	19 (1.0)	13 (0.9)	2 (0.1)	6 (3.0)	0
		amphotericin B liposome	5 (0.1)	1 (0.2)	5 (0.3)	3 (0.2)	0	1 (0.5)	1 (0.1)

Note:^a^ including 54 cases of “Prophylaxis + Therapy”

### Antibiotic use in four types of pediatric wards

There were significant differences between the proportions of patients receiving antibiotics in the general pediatric medical wards (65.1%, *n* = 985), the pediatric surgical wards (53.9%, *n* = 771), the PICU (84.0%, *n* = 168) and the neonatal wards (74.2%, *n* = 617); (χ^2^ = 136.315, *P* < 0.001). There was no significant difference of antibiotic use rate between boys and girls in the four types of wards (neonatal wards: boys 76.7%, girls 71.3%, χ^2^ = 3.171, *P* = 0.075; PICU: boys 85.3%, girls 81.7% χ^2^ = 0.437, *P* = 0.509; general pediatric medical wards: boys 63.6%, girls 67.1%, χ^2^ = 2.085, *P* = 0.149; pediatric surgical wards: boys 53.7%, girls 54.4%, χ^2^ = 0.043, *P* = 0.836). In the general pediatric medical wards, 40 different antibiotics were recorded of which cefuroxime was the most often prescribed (17.0%, *n* = 257) followed by ceftriaxone (16.8%, *n* = 255), azithromycin (12.9%, *n* = 195), amoxicillin/clavulanic acid (6.9%, *n* = 104) and meropenem (5.9%, *n* = 89). In pediatric surgical wards, 34 different antibiotics were recorded, of which cefuroxime was the most often prescribed (21.4%, *n* = 306) followed by ornidazole (10.8%, *n* = 154), ceftriaxone (7.8%, *n* = 111), cefoperazone/sulbactam (7.3%, *n* = 105) and metronidazole (6.2%, *n* = 89). In the treatment of children in PICU, 30 different antibiotics were recorded, of which meropenem was the most often prescribed (23.5%, *n* = 47) followed by cefoperazone/sulbactam (23.0%, *n* = 46), cefuroxime (15.0%, *n* = 30), ampicillin/sulbactam (14.5%, *n* = 29) and azithromycin (11.0%, *n* = 22). In neonatal wards, 28 different antibiotics were recorded, of which cefotaxime was the most often prescribed (37.3%, *n* = 310) followed by ampicillin/sulbactam (20.2%, *n* = 168), meropenem (13.8%, *n* = 115), ticarcillin/clavulanic acid (7.6%, *n* = 63) and metronidazole (2.9%, *n* = 24) (Table 2).

### Indications for antibiotic treatment

Of all children on antibiotics, 74.5% received antibiotics for antimicrobial therapy, 23.4% received antibiotics for prophylactic treatment and 2.1% as prophylaxis plus therapy. The five most commonly used generic antibiotics for prophylaxis were cefuroxime (45.8%, *n* = 297), ornidazole (13.9%, *n* = 90), ceftriaxone (12.5%, *n* = 81), metronidazole (9.0%, *n* = 58) and cefoperazone/sulbactam (8.3%, *n* = 54). The five most commonly used generic antibiotics for therapy were cefotaxime (17.7%, *n* = 344), ceftriaxone (16.2%, *n* = 315), cefuroxime (15.6%, *n* = 304), meropenem (15.3%, *n* = 298) and azithromycin (11.3%, *n* = 220).

Prophylaxis as an indication for antimicrobial use in the general pediatric medical, PICU, and neonatal wards was rare (2.3, 4.2 and 6.3%, respectively), whereas in the pediatric surgical wards prophylactic antibiotic treatment accounted for 68.1% of prescriptions. In addition, 30% of the children in pediatric surgical wards receiving antibiotics received two or more types of antibiotic prophylaxis. The majority (63.5%) of patients receiving antibiotic treatment in the pediatric surgical wards were prescribed two or more types of antibiotics whereas far fewer (36.4%) received a single antibiotic. One half of children in PICU and approximately 30% in the general pediatric medical and neonatal wards received two or more types of antibiotics (Table 3).

**Table 3:** Indications (therapy, prophylaxis) and combinations of antibiotics used in the four types of pediatric wards

Indications	Number of Participants (*n* = 2541)	General pediatric medical wards (n = 985)	Pediatric surgical wards (n = 771)	PICU (n = 168)	Neonatal wards (n = 617)
**Prophylaxis**	**594 (23.4)**	**23 (2.3)**	**525 (68.1)**	**7 (4.2)**	**39 (6.3)**
*Single*	*427 (71.9)*	*20 (87.0)*	*366 (69.7)*	*6 (85.7)*	*35 (89.7)*
*Two antibiotics combination*	*161 (27.1)*	*1 (4.3)*	*156 (29.7)*	*1 (14.3)*	*3 (7.7)*
*Three antibiotics combination*	*6 (1.0)*	*2 (8.7)*	*3 (0.6)*	*0*	*1 (2.6)*
**Therapy**	**1893 (74.5)**	**960 (97.5)**	**214 (27.8)**	**156 (92.8)**	**563 (91.2)**
*Single*	*1215 (64.2)*	*667 (69.5)*	*78 (36.4)*	*78 (50.0)*	*392 (69.6)*
*Two antibiotics combination*	*607 (32.1)*	*257 (26.8)*	*124 (57.9)*	*63 (40.4)*	*163 (29.0)*
*Three antibiotics combination*	*71 (3.7)*	*36 (3.7)*	*12 (5.6)*	*15 (9.6)*	*8 (1.4)*
**Prophylaxis + Therapy**	**54 (2.1)**	**2 (0.2)**	**32 (4.1)**	**5 (3.0)**	**15 (2.4)**
*Single*	*27 (50.0)*	*0*	*14 (43.8)*	*0*	*13 (86.7)*
*Two antibiotics combination*	*20 (37.0)*	*0*	*18 (56.2)*	*1 (20.0)*	*1 (6.7)*
*Three antibiotics combination*	7 (13.0)	2 (100.0)	0	4 (80.0)	1 (6.7)

## Discussion

Antimicrobial treatment is particularly high for hospitalized pediatric patients owing to both community- and healthcare-associated infections and nonspecific disease presentations which cause difficulty in excluding bacterial infections. In our study using seven one-day, annually-spaced PPSs in a single tertiary care center, a total of 63.9% of hospitalized children received at least one dose of an antibiotic. This proportion is slightly lower than the results of a one-day PPS performed by another group on antibiotic treatment of hospitalized children across nine provinces in China (67.76%) [16] but much higher than the rate of antibiotic prescription in a tertiary children’s hospital surveyed in Sichuan, China (46.1%) [17]. Compared with Western countries (Sweden 35.5% [18], Norway 24% [19], UK 40.9% [20], Italy 38.9% [21]), our study revealed a much higher rate of antibiotic use in China indicating that the use of antibiotics in the treatment of hospitalized children varies by countries and regions. Thus, there is a need to collect and analyze data regarding the use of antibiotics in local regions and even individual hospitals in order to tailor pediatric antibiotic management more effectively. The proportion of patients receiving antibiotics in neonatal wards (74.2%) was much higher than in the general pediatric medical wards (65.1%) and pediatric surgical wards (53.9%). This is not surprising since, unlike children and adults, neonates often exhibit nonspecific clinical and laboratory signs making it difficult to distinguish infections from other disease processes. Furthermore, because of immature immune systems and the risk for serious complications, neonates often receive antibiotics before the results of microbiological testing are available. Therefore, improving diagnostics plays an important role in improving the effective use of antibiotics. In a study from the United States, Cantey et al. found that only a small fraction of antibiotic use in the NICU was directed toward proven infection [22]. These results may reflect the challenges of diagnosing infections in children resulting in a high rate of empirical antibiotic use, particularly in the treatment of neonates.

Consistent with most study results [17, 21, 23–25], our analysis shows that third-generation cephalosporin was the most commonly prescribed antibiotic in this study population. In principle, the use of broad-spectrum antibiotics is caused by a concern regarding the possibility of bacterial etiology. Since bacterial culture results are often not available for 24–72 h, the initial treatment for infection is often empirical. Weiss and colleagues revealed that delayed antimicrobial therapy was an independent risk factor for mortality and prolonged organ dysfunction in pediatric sepsis [26]. Due to the delay in laboratory test reports and prior treatment with antibiotics which may have an important impact on the ability to curb the severity of disease, a common approach is to prescribe broad-spectrum antimicrobial agents or combinations of antibiotics for pediatric patients. However, the use of third-generation cephalosporins has been associated with the development of resistant pathogens [12, 13]. Therefore, they should always be used cautiously.

In this study, carbapenems ranked third in total antimicrobial treatment of pediatric patients and was almost totally represented by meropenem. Meropenem was among top five antibiotics prescribed in the general pediatric, PICU and neonatal wards and sixth in the pediatric surgical wards. Carbapenems are often considered to be last resort agents reserved for the treatment of infections highly resistant to most antimicrobial treatments. Unfortunately, whether it is in the literature [16] or in clinical practice, the empirical use of third-generation cephalosporins in children is so common in China that pediatricians are advised to prescribe carbapenems only to severely infected children after admission. Troublingly, this phenomenon does not exist only in China. A survey conducted in 226 hospitals in 41 Asian countries indicated that meropenem was widely prescribed to neonates [15]. Studies have shown that prior exposure to carbapenems is an independent risk factor for acquiring carbapenem-resistant, gram-negative bacterial infections [12, 14]. A recent study revealed that the prevalence of carbapenem-resistant Enterobacteriaceae strains in children has been increasing annually in China [27]. Therefore, it is recommended that the prescription of meropenems should be strictly controlled and monitored to effectively limit the emergence of antimicrobial resistance. In the current “information age”, a clinical decision support system (CDSS) embedded in an electronic health record could be a useful tool for monitoring and management of antimicrobial treatment. In fact, it has been confirmed that a CDSS had a substantial impact on the overall prescribing of broad-spectrum antibiotics in the treatment of both pediatric and adult patients with acute respiratory infections in a primary care setting [28].

Of all children on antibiotics in this study, 23.4% received prophylactic treatment, and low prescribing rates of antibiotic prophylaxis were observed in the general pediatric medical (2.3%), PICU (4.2%) and neonatal wards (6.3%). This was a reassuring finding, since prophylactic use of antibiotics to prevent infections is controversial. The results of the current study did not support the administration of antibiotics for surgical prophylaxis as a potential quality indicator. Prophylaxis in pediatric surgical wards, however, accounted for 68.1% of antibiotic prescriptions. This was much higher than the percentage of antibiotic prophylaxis for surgical diseases in the treatment of children in Europe (26.6%) [29]. Also, 30% of the children in surgical wards in this study received two or more types of antibiotic prophylaxis. The most common surgeries with antibiotic prophylaxis were head and neck surgeries through the oropharyngeal mucosa, hepatobiliary system surgeries, colorectal surgeries, and hypospadias repair. A second-generation cephalosporin combined with nitroimidazoles was the most commonly used antibiotic prophylaxis for these surgeries, which explains the ranking of cefuroxime and ornidazole usage for prophylaxis as firstly and secondly in our study. Nevertheless, our study indicates that antibiotic prophylaxis is one of the most common indications for antibiotic prescribing in the pediatric population. Therefore, measures should be taken to improve antibiotic management for surgical prophylaxis. For example, the hospital information system can be set to automatically notify surgeons in the doctor workstation when they attempt to prescribe inappropriate prophylactic antibiotics.

There are several strengths to this study. Firstly, all wards were investigated, so our research population included medical and surgical wards with a broad spectrum of diseases. Furthermore, the wards were divided into pediatric medical, PICU, neonatal wards and pediatric surgical wards, which would help to manage antibiotic use based on the unique challenges in the different wards. This distinction was rare in previous studies. Secondly, seven repeated PPSs were conducted on all wards within the same hospital so that the selection bias would be reduced and the overall prescribing of antibiotics in our hospital would be better reflected. There were also certain limitations in our study. Firstly, one of the drawbacks of PPS is that it cannot be judged whether the purpose of using antibiotics at this point is empirical or targeted medication. Secondly, in this study, all PPSs were conducted in autumn and winter, which could not reflect the fact that antibiotic use may change according to seasons of the year. Thirdly, the study was conducted at a single healthcare center, and the results may or may not be able to be extrapolated to other similar hospitals. Nevertheless, as a tertiary children’s hospital in China, patients come from multiple regions of the country. Thus, the results in this study do reflect the use of antibiotics in the treatment of hospitalized children.

## Conclusion

This study has revealed the high rate of overall antibiotic use in the treatment of children, and most particularly neonates and children requiring surgery, in a tertiary hospital in Shanghai. The most commonly prescribed antibiotics were third-generation cephalosporins. Meropenem was among top five antibiotics prescribed in the general pediatric, PICU and neonatal wards and sixth in the pediatric surgical wards. The prescription of these drugs in children should be strictly controlled and monitored to effectively limit the development of antimicrobial resistance. Additionally, measures should be taken to improve the management of antibiotics for surgical prophylaxis in children. The CDSS is one tool, already deemed useful in the literature, for the management of antimicrobial treatment in children and adults.

## Data Availability

The datasets used and analyzed during the current study are available from the corresponding author on reasonable request.

## Abbreviations

PPS: Point prevalence survey
PICU: Pediatric intensive care unit
NICU: Neonatal intensive care unit
CDSS: Clinical decision support system

## References

[1] Li Y, Xu J, Wang F, Wang B, Liu L, Hou W (2012). Overprescribing in China, driven by financial incentives, results in very high use of antibiotics, injections, and corticosteroids. Health Aff.

[2] Wang J, Sheng Y, Ni J, Zhu J, Zhou Z, Liu T (2019). Shanghai parents’ perception and attitude towards the use of antibiotics on children: a cross-sectional study. Infect Drug Resist.

[3] Xu Y, Lu J, Sun C, Wang X, Hu YJ, Zhou X (2020). A cross-sectional study of antibiotic misuse among Chinese children in developed and less developed provinces. J Infect Dev Ctries.

[4] Sun C, Hu YJ, Wang X, Lu J, Lin L, Zhou X (2019). Influence of leftover antibiotics on self-medication with antibiotics for children: a cross-sectional study from three Chinese provinces. BMJ Open.

[5] Xia R, Hu X, Willcox M, Li X, Li Y, Wang J (2019). How far do we still need to go? A survey on knowledge, attitudes, practice related to antimicrobial stewardship regulations among Chinese doctors in 2012 and 2016. BMJ Open.

[6] Quan-Cheng K, Jian-Guo W, Xiang-Hua L, Zhen-Zhen L (2016). Inappropriate use of antibiotics in children in China. Lancet.

[7] Dong F, Zhang Y, Yao K, Lu J, Guo L, Lyu S (2018). Epidemiology of Carbapenem-resistant *Klebsiella pneumoniae* bloodstream infections in a Chinese Children's hospital: predominance of New Delhi Metallo-Lactamase-1. Microb Drug Resist.

[8] Anandalwar SP, Milliren C, Graham DA, Hills-Dunlap JL, Kashtan MA, Newland J (2020). Trends in the use of surgical antibiotic P\prophylaxis in general pediatric surgery: are we missing the mark for both stewardship and infection prevention?. J Pediatr Surg.

[9] Schultz L, Lowe TJ, Srinivasan A, Neilson D, Pugliese G (2014). Economic impact of redundant antimicrobial therapy in US hospitals. Infect Control Hosp Epidemiol.

[10] van Houten CB, Cohen A, Engelhard D, Hays JP, Karlsson R, Moore E (2019). Antibiotic misuse in respiratory tract infections in children and adults—a prospective, multicentre study (TAILORED treatment). Eur J Clin Microbiol Infect Dis.

[11] Yusuf Y, Murni IK, Setyati A (2017). Irrational use of antibiotics and clinical outcomes in children with pneumonia. Pediatrica Indonesiana.

[12] Clock SA, Ferng Y, Tabibi S, Alba L, Patel SJ, Jia H (2017). Colonization with antimicrobial-resistant gram-negative bacilli at neonatal intensive care unit discharge. J Pediatric Infect Dis Soc.

[13] Bharadwaj R, Robinson ML, Balasubramanian U, Kulkarni V, Kagal A, Raichur P (2018). Drug-resistant Enterobacteriaceae colonization is associated with healthcare utilization and antimicrobial use among in patients in Pune, India. BMC Infect Dis.

[14] Sahbudak BZ, Bekmezci N, Soylu M, Sen S, Avcu G, Aydemir S (2018). The prospective evaluation of risk factors and clinical influence of carbapenem resistance in children with gram-negative bacteria infection. Am J Infect Control.

[15] Versporten A, Bielicki J, Drapier N, Sharland M, Goossens H (2016). The worldwide antibiotic resistance and prescribing in European children (ARPEC) point prevalence survey: developing hospital-quality indicators of antibiotic prescribing for children. J Antimicrob Chemother.

[16] Zhang J, Liu G, Zhang W, Shi H, Lu G, Zhao C (2018). Antibiotic usage in Chinese children: a point prevalence survey. World J Pediatr.

[17] Miao R, Wan C, Wang Z, Zhu Y, Zhao Y, Zhang L (2020). Inappropriate antibiotic prescriptions among pediatric in patients in different type hospitals. Medicine.

[18] Luthander J, Bennet R, Nilsson A, Eriksson M (2019). Antimicrobial use in a Swedish pediatric hospital: results from eight point-prevalence surveys over a 15-year period (2003-2017). The Pediatr Infect Dis J.

[19] Thaulow CM, Berild D, Eriksen BH, Myklebust TÅ, Blix HS (2019). Potential for more rational use of antibiotics in hospitalized children in a country with low resistance - data from eight point prevalence surveys. Pediatr Infect Dis J.

[20] Gharbi M, Doerholt K, Vergnano S, Bielicki JA, Paulus S, Menson E (2016). Using a simple point-prevalence survey to define appropriate antibiotic prescribing in hospitalized children across the UK. BMJ Open.

[21] De Luca M, Donà D, Montagnani C, Lo Vecchio A, Romanengo M, Tagliabue C (2016). Antibiotic prescriptions and prophylaxis in Italian children. Is it time to change? Data from the ARPEC project. PLoS One.

[22] Cantey JB, Wozniak PS, Sánchez PJ (2015). Prospective surveillance of antibiotic use in the neonatal intensive care unit: results from the SCOUT study. Pediatr Infect Dis J.

[23] Yoshida S, Takeuchi M, Kawakami K (2018). Prescription of antibiotics to pre-school children from 2005 to 2014 in Japan: a retrospective claims database study. J Public Health.

[24] Labi A, Akufo C, Bjerrum S, Owusu E, Enweronu-Laryea C, Opintan JA (2018). Antibiotic prescribing in pediatric inpatients in Ghana: a multi-Centre point prevalence survey. BMC Pediatr.

[25] Krasniqi S, Versporten A, Jakupi A, Raka D, Daci A, Krasniqi V (2017). Antibiotic utilization in adult and children patients in Kosovo hospitals. Eur J Hosp Pharm.

[26] Weiss SL, Fitzgerald JC, Balamuth F, Alpern ER, Lavelle J, Chilutti M (2014). Delayed antimicrobial therapy increases mortality and organ dysfunction duration in pediatric sepsis. Crit Care Med.

[27] Guo Y, Hu FP, Zhu DM, Wang CQ, Wang AM, Zhang H (2018). Antimicrobial resistance changes of carbapenem - resistant Enterobacteriaceae strains isolated from children. Zhonghua Er Ke Za Zhi.

[28] Mainous AG, Lambourne CA, Nietert PJ (2013). Impact of a clinical decision support system on antibiotic prescribing for acute respiratory infections in primary care: quasi- experimental trial. J Am Med Inform Assoc.

[29] Hufnagel M, Versporten A, Bielicki J, Drapier N, Sharland M, Goossens H (2019). High rates of prescribing antimicrobials for prophylaxis in children and neonates: results from the antibiotic resistance and prescribing in European children point prevalence survey. J Pediatric Infect Dis Soc.

